# Mass Spectrometry-Based Profiling of Histone Post-Translational Modifications in Uveal Melanoma Tissues, Human Melanocytes, and Uveal Melanoma Cell Lines – A Pilot Study

**DOI:** 10.1167/iovs.65.2.27

**Published:** 2024-02-13

**Authors:** Martina C. Herwig-Carl, Amit Sharma, Verena Tischler, Natalie Pelusi, Karin U. Loeffler, Frank G. Holz, Michael Zeschnigk, Solange Landreville, Claudia Auw-Haedrich, Roberta Noberini, Tiziana Bonaldi

**Affiliations:** 1 Department of Ophthalmology, University Hospital Bonn, Bonn, Germany; 2 Division of Ophthalmic Pathology, University Hospital Bonn, Bonn, Germany; 3 Centrum for Integrated Oncology Aachen Bonn Cologne Düsseldorf (CIO ABCD), Germany; 4 Department of Neurosurgery, University Hospital Bonn, Bonn, Germany; 5 Institute of Pathology, University Hospital Bonn, Bonn, Germany; 6 Institute of Human Genetics, University Hospital Essen, Essen, Germany; 7 Department of Ophthalmology and Otolaryngology-Cervicofacial Surgery, Université Laval, Quebec City, Quebec, Canada; 8 Department of Ophthalmology, University Hospital Freiburg, Freiburg, Germany; 9 Department of Experimental Oncology, European Institute of Oncology (IEO) IRCCS, Milan, Italy; 10 Department of Oncology and Haematology-Oncology (DIPO), University of Milan, Milan, Italy

**Keywords:** uveal melanoma (UM), histone post-translational modifications (PTMs), cell lines, melanocytes, tumor heterogeneity

## Abstract

**Purpose:**

Epigenetic alterations in uveal melanoma (UM) are still neither well characterized, nor understood. In this pilot study, we sought to provide a deeper insight into the possible role of epigenetic alterations in the pathogenesis of UM and their potential prognostic relevance. To this aim, we comprehensively profiled histone post-translational modifications (PTMs), which represent epigenetic features regulating chromatin accessibility and gene transcription, in UM formalin-fixed paraffin-embedded (FFPE) tissues, control tissues, UM cell lines, and healthy melanocytes.

**Methods:**

FFPE tissues of UM (*n* = 24), normal choroid (*n* = 4), human UM cell lines (*n* = 7), skin melanocytes (*n* = 6), and uveal melanocytes (*n* = 2) were analyzed through a quantitative liquid chromatography-mass spectrometry (LC-MS) approach.

**Results:**

Hierarchical clustering showed a clear separation with several histone PTMs that changed significantly in a tumor compared to normal samples, in both tissues and cell lines. In addition, several acetylations and H4K20me1 showed lower levels in BAP1 mutant tumors. Some of these changes were also observed when we compared GNA11 mutant tumors with GNAQ tumors. The epigenetic profiling of cell lines revealed that the UM cell lines MP65 and UPMM1 have a histone PTM pattern closer to the primary tissues than the other cell lines analyzed.

**Conclusions:**

Our results suggest the existence of different histone PTM patterns that may be important for diagnosis and prognosis in UM. However, further analyses are needed to confirm these findings in a larger cohort. The epigenetic characterization of a panel of UM cell lines suggested which cellular models are more suitable for epigenetic investigations.

Uveal melanoma (UM) is the most frequent intraocular primary malignancy in Caucasian adults.^1^^,^^2^ It significantly differs from skin melanoma by its clinical behavior, its genetic signature, as well as its responsiveness to various therapies.^3^ In about 50% of patients, UM metastasizes hematogenously and most often to the liver.^4^ Driver mutations in UM are found mostly in the *GNAQ*, *GNA11*, *EIF1AX*, *SF3B1*, and *BAP1* genes.^5^^–^^9^ GNAQ and GNA11 bear initiating mutations that occur in up to 90% of UM in a mutually exclusive manner.^9^^,^^10^ An inactivating mutation of BRCA1-associated protein 1 (BAP1) – a tumor suppressor gene – usually occurs in later stages and is highly associated with metastatic disease in UM.^5^ As an ubiquitin carboxy-terminal hydrolase, BAP1 is a member of the Polycomb Repressive-Deubiquitinase complex, which interacts with Polycomb Repressive Complexes 1 and 2, thus exerting indirect effects on histone H3K27 methylation levels.^11^ The BAP1 status as an important prognostic factor for metastasis in UM can be determined by next generation sequencing (NGS) as well as by immunohistochemistry (IHC)^12^^–^^14^ (which has been shown to yield a reliable staining reaction but can be of limited reliability in archival tissue^15^).

Although genetic and chromosomal alterations are well studied in UM, there is an unmet need in this tumor for further investigation of epigenetic features, which frequently occur during cancer development and progression.^16^^–^^20^ In contrast to genetic changes, epigenetic modifications are not permanent and can be reverted by using epigenetic drugs.^19^^,^^20^ Histone post-translational modifications (PTMs) are epigenetic features that regulate the chromatin structure and gene transcription.^21^ Under physiological conditions, there is a dynamic balance in the levels and activities of the different proteins that can write, read, and remove histone PTMs (the so-called histone writers, readers, and erasers).^22^ However, an imbalance of histone writers and erasers, due to mutations, amplifications, or aberrant regulation of their expression, is often observed in cancer, leading to altered histone PTM patterns and, in turn, aberrant gene expression profiles, with possible activation of oncogenic drivers and pathways or silencing of tumor suppressor genes. For instance, in UM, the transcriptional silencing of MHC2TA has been associated with high levels H3K27me3,^23^ whereas overexpression of the transcription factor HES1, which is linked to metastatic capacity, has been described to depend on the presence of H3K4me3 at the *HES1* gene promoter.^24^ Furthermore, there is evidence that histone modifiers, in particular histone deacetylases (HDACs), have an impact on UM cell differentiation in vitro^25^ and in vivo (animal models).^26^^–^^28^ Furthermore, aberrant levels of histone PTMs have been identified as diagnostic and prognostic histone PTM markers in different types of cancers.^18^ However, a comprehensive histone PTM profiling in UM – to the best of our knowledge – has not been performed yet.

In recent years, quantitative mass spectrometry (MS) has emerged as the most suitable tool for the unbiased, comprehensive, and quantitative analysis of histone PTMs in biological samples, including patient-derived tissues.^18^^,^^29^

In this pilot study, histone PTMs have been comprehensively analyzed in human formalin-fixed paraffin-embedded tissues (FFPE) from UM tissues and control choroids, as well as in different human UM cell lines and normal melanocytes (from skin and uvea), identifying histone marks linked with BAP1 status and tumor staging and grading. This study represents the initial step toward a deeper understanding of the possible involvement of epigenetic mechanisms in the pathogenesis of UM and the identification of epigenetic prognostic markers for UM, which may serve as biomarkers in the future.

## Methods

The UM tissues (*n* = 24) and human choroids (*n* = 4) as well as human UM cell lines (*n* = 7), human skin melanocytes (*n* = 6), and uveal melanocytes (*n* = 2) were analyzed by established quantitative mass spectrometry protocols^30^ for 48 (tissue) or 58 (cell lines) histone 3 and histone 4 PTMs.

### Tissue Sample Selection and Collection

UM tissues were collected from the archives of the ophthalmic pathology laboratory of the University Eye Hospital Bonn and Freiburg, Germany. The research was conducted in adherence to the tenets of the Declaration of Helsinki. Ethic Board Approval of the University of Bonn (No. 328/16) and Freiburg (No. 21-1356) was obtained. The tumors were graded according to the TNM cancer staging system (T2: *n* = 8, T3: *n* = 13, T4: *n* = 3; G1: *n* = 5, G2: *n* = 12, and G3: *n* = 7) of the 8th edition of American Joint Committee on Cancer (AJCC) Staging Manual.^31^ Grade 1 (G1) refers to spindle (> 90% spindle cells), grade 2 (G2) to mixed (> 10% epithelioid cells and < 90% spindle cells), and grade 3 (G3) to epithelioid (> 90% epithelioid cells) morphology of the primary tumor. The control choroid was harvested from enucleated globes with an unaffected posterior segment and no history of uveal melanoma or another ocular tumor. The characteristics of the investigated UM and the control choroid are shown in Table 1.

**Table 1.** Characteristics of Analyzed Human Tissues

	Age, y	Gender, F/M	T Stage	Grade	BAP1 IHC	BAP1 NGS	Driver Mutations
Control uvea #1	62	M	N/A	N/A	N/A	N/A	N/A
Control uvea #2	57	M	N/A	N/A	N/A	N/A	N/A
Control uvea #3	79	F	N/A	N/A	N/A	N/A	N/A
Control uvea #4	71	M	N/A	N/A	N/A	N/A	N/A
UM1	74	F	T3a	G3	UKN	Mut	GNAQ
UM2	68	M	T2c	G3	Mut	UKN	UKN
UM3	80	F	T2a	G2	WT	WT	GNAQ
UM4	64	M	T3a	G2	UKN	Mut	GNA11
UM5	44	F	T3b	G2	UKN	Mut	GNA11
UM6	81	F	T3a	G2	UKN	WT	EIF1AX
UM7	81	F	T4b	G1	UKN	Mut	GNA11
UM8	52	M	T3a	G1	UKN	UKN	UKN
UM9	72	F	T2c	G2	UKN	Mut	GNA11
UM10	79	F	T3a	G1	UKN	WT	GNA11
							SF3B1
UM11	63	M	T4b	G3	UKN	Mut	GNA11
UM12	82	M	T4e	G3	Mut	Mut	GNA11
UM13	83	M	T2b	G3	Mut	Mut	GNAQ
UM14	58	M	T2a	G1	WT	WT	GNAQ
UM15	52	M	T3a	G2	Mut	Mut	CYSLTR2
UM16	85	M	T3a	G2	WT	WT	GNAQ
							SF3B1
UM17	74	F	T2a	G2	WT	WT	GNAQ
UM18	82	M	T3a	G2	Mut*	WT	GNA11
UM19	56	M	T3a	G1	Mut*	WT	GNAQ
UM20	52	M	T3c	G2	Mut*	WT	GNAQ
							EIF1AX
UM21	73	F	T2a	G3	WT	WT	GNAQ
							EIF1AX
UM22	58	F	T2a	G2	WT^*^	Mut	PLCB4
UM23	68	F	T3a	G3	UKN	WT	GNAQ
							EIF1AX
UM24	87	F	T3a	G2	Mut	Mut	GNA11

F, female; IHC, immunohistochemistry; M, male; Mut, mutation; NGS, next generation sequencing; UKN, unknown; UM, uveal melanoma; WT, wild type.

* Differences between BAP1 status determined by IHC and NGS.

### BAP1 Immunohistochemical Staining

Immunohistochemical staining for the BAP1 protein was performed using the labeled streptavidin-biotin (LSAB) staining method. In detail, sections were cut from FFPE tissue blocks at a thickness of 5 µm and mounted onto Superfrost glass slides. Sections were deparaffinized and rehydrated. Heat-mediated antigen retrieval was performed in a microwave (750 W) with citrate buffer (pH 6.0) for 10 minutes followed by cooling down for 30 minutes; application of klear dual enzyme block (E36-18; GBI Labs, Bothell, WA, USA) for 15 minutes at room temperature (RT); washing for 15 minutes (RT) with TBST (Tris buffer and Tween 20); application of protein block solution (DAKO X0909) for 10 minutes at RT; incubation with the primary antibody (rabbit anti-BAP1, Invitrogen PA5-105741, dilution 1:150 using the antibody diluent [DAKO S0809]) overnight at 4°C and 1 hour at 37°C; incubation with Envision Flex+ Rabbit Linker (DAKO K8019) for 15 minutes followed by incubation with the secondary antibody (Dako EnVision Dual Link System HRP, K4063, Lot 10101679) for 60 minutes at RT. The specimens were then stained with AEC (3-amino-9-ethylcarbazole, DAKO K3469) for 10 minutes and counterstained with Mayer's hemalum.

### Analysis of BAP1, GNAQ, and GNA11 Status by Next Generation Sequencing

Because the BAP1 status could not be reliably determined by IHC in all specimens due to the archival nature of the tissues, NGS of extracted tumor DNA from FFPE tissues was used for the determination of *BAP1* mutations and the *GNAQ / GNA11* status which were available for 12 specimens (12 specimens did not yield sufficient DNA for the analysis). In brief, for nucleic acid extraction from FFPE material, the tumor area was first macrodissected from histological sections. For DNA extraction, tissues were lysed overnight at 56°C. DNA and RNA were then purified using the Maxwell RSC DNA or RNA FFPE Kits (Promega) according to manufacturer's recommendations, and eluted in 120 µL and 50 µL of nuclease-free water, respectively. The nucleic acid concentration was determined on a Quantus fluorometer using the QuantiFluor ONE ds DNA System (Promega) and the QuantiFluor RNA System (Promega). Generation of the sequencing library was performed using a QIAseq Pan-cancer Multimodal panel (Qiagen) with an input of 40 ng DNA and 150 ng RNA according to the manufacturers protocol. Amplification products were subjected to NGS on a NextSeq 550 sequencing platform (Illumina). Sequencing data were analyzed for genomic variants and fusion genes using the CLC Genomics Workbench/Server 22/23 (Qiagen Bioinformatics). Variants were manually filtered for artifacts, silent variants, and assumed germline variants, and relevant somatic variants with an alternative allele frequency ≥5% were reported. For classification and interpretation of somatic variants the following databases were used: dbSNP, ClinVar, and Mastermind Genomic Search Engine (https://www.genomenon.com/mastermind) in their respective current versions.

### Cells

The characteristics of the melanocytes and the UM cell lines are listed in Table 1. Human primary skin melanocytes were harvested from tissues removed during blepharoplasty surgery (ethics approval granted by the ethics committee of the University Hospital Bonn, # 25/19) using a protocol described by D. Bennett.^32^ The uveal melanocytes were kindly provided by S. Landreville (ethics approval granted by the ethics committee of the Centre de recherche du CHU de Québec-Université Laval, #2021-5273 and #2012-1483).^33^^,^^34^ The human UM cell lines^35^ were kindly provided by M. Zeschnigk (UPMD1,^36^ UPMD2,^36^ and UPMM1^37^), received from the laboratory in Essen, Germany (92.1^38^ and Mel 202^39^) or purchased from the American Type Culture Collection (ATCC; MP38 [ATCC CRL-3296],^40^^,^^41^ and MP65 [ATCC CRL-3299]^40^). A retinal pigment epithelium (RPE) cell line (ARPE19) was used as an additional control.

### Analysis of Histone Post-Translational Modifications by Mass Spectrometry

Histones were extracted from 5 to 10 tissue sections of 10 µm thick or 2 million cells as previously described.^42^ Approximately 4 µg of histone octamer were mixed with an equal amount of heavy-isotope labeled histones, which were used as an internal standard,^43^ and separated on a 17% SDS-PAGE gel. Histone bands were excised, chemically acylated with propionic anhydride and in-gel digested with trypsin, followed by peptide N-terminal derivatization with phenyl isocyanate (PIC).^30^ Peptide mixtures were separated by reversed-phase chromatography on an EASY-Spray column (Thermo Fisher Scientific, Fair Lawn, NJ, USA), 25-cm long (inner diameter 75 µm, PepMap C18, and 2 µm particles), which was connected online to a Q Exactive Plus instrument (Thermo Fisher Scientific) through an EASY-Spray Ion Source (Thermo Fisher Scientific), as described.^30^ The acquired RAW data were analyzed using EpiProfile 2.0,^44^ followed by manual validation. For each histone modified peptide, a percentage relative abundance (%RA) value for the sample (light channel - L) or the internal standard (heavy channel - H) was estimated by dividing the area under the curve of each acetylated peptide for the sum of the areas corresponding to all the observed forms of that peptide and multiplying by 100. Light/Heavy (L/H) ratios of %RA were then calculated and are reported in Supplementary Table S1. Data analysis and visualization, and statistical tests were performed using Perseus^45^ and GraphPad Prism. The MS data have been deposited to the ProteomeXchange Consortium^46^ via the PRIDE partner repository with the dataset identifier PXD043551. The statistical tests used are mentioned in the Figure legends for each analysis.

## Results

### FFPE Tumor Tissues Show Differential Histone PTM Patterns Compared to Healthy Choroids

**Figure 1.**
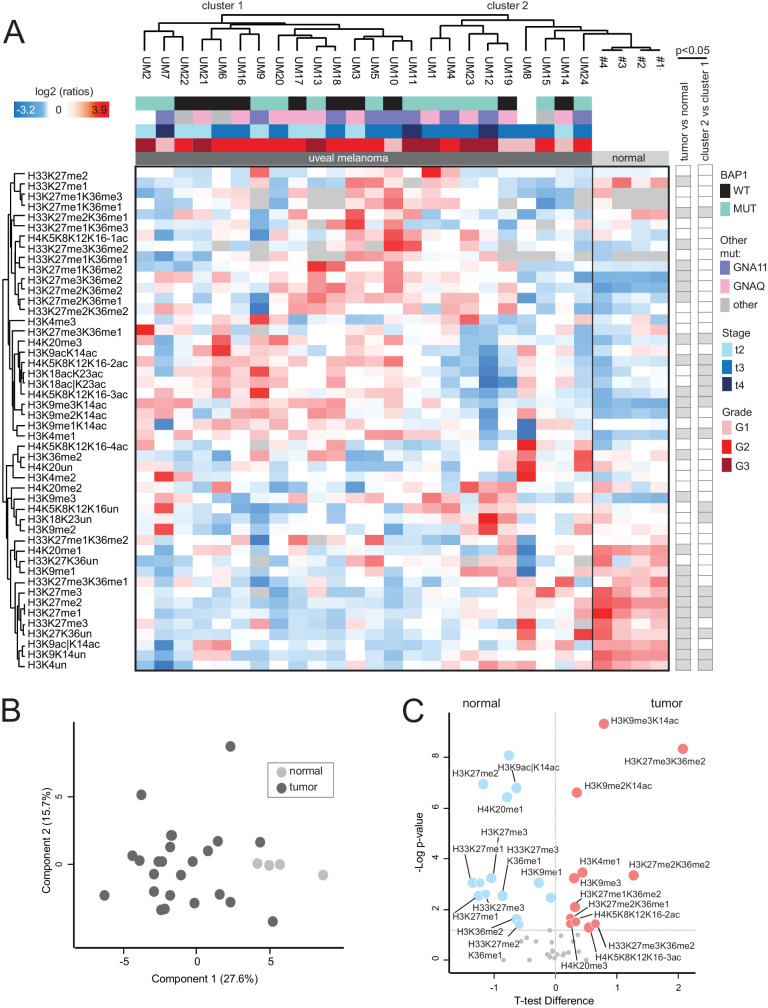
**Histone PTM profiles in uveal melanoma (UM) and normal choroid.** (**A**) Hierarchical clustering based on histone PTM levels in normal choroids and UM tissues. Light/heavy (L/H) relative abundances ratios were obtained using a spike-in strategy (light channel: sample and heavy channel: spike-in standard), and were normalized over the average ratios across samples. Stage, grade (according to the 8th edition AJCC staging manual) and BAP1 status are indicated for tumor samples. The *grey color* indicates peptides that were not quantified. The *right panels* highlight significant changes (*P* < 0.05 by Welch's *T*-test) for the comparison of normal and tumor tissues, and tumor cluster 1 versus tumor cluster 2. (**B**) Principal component analysis (PCA) based on histone PTM data obtained from the samples shown in **A**. (**C**) Volcano-plot showing significantly up- (*red*) or down- (*blue*) regulated histone PTMs in UM (tumor) compared to normal choroids (normal). *P* < 0.05 by Welch's *T*-test. The symbol “|” indicates that the modification is present on only one of the indicated residues.

We quantified histone H3 and H4 lysine methylations and acetylations from 24 UM primary FFPE tissues and 4 human choroid samples using established quantitative MS protocols.^18^^,^^30^ Because age also has an impact on epigenetic changes, melanocytes and tumor tissues from patients of similar age were analyzed (see Table 1). Up to 48 differentially modified peptides were analyzed from the archival FFPE UM tissues. Of note, some modifications (e.g. methylation of H3K79 and H3K18) were not quantified, because they were previously shown to be altered by FFPE storage.^29^ Hierarchical clustering based on histone PTM levels showed obvious differences between the control uvea from enucleated eyes with a normal posterior segment and UM tissues (Fig. 1A). This finding was confirmed by principal component analysis (PCA) in which all UMs (except for UM 24) clustered away from the control choroid (Fig. 1B). Several histone PTMs were significantly up- or downregulated in tumor tissues compared to control uvea (Fig. 1C). Modifications showing decreased levels in tumors included histone H3 and H3.3 H3K27 methylations, H3K36me1/me2, H3K9ac|K14ac (a peptide carrying an acetyl group on H3K9 or H3K14), and H4K20me1. Modifications increased in tumors were H3K4me1, the H3K9me3-containing peptides, histone H3 27-40 peptides containing K36me2 in combinations with K27 methylations, the multiply acetylated histone H4 tail and H4K20me3.

### Subgroup Analysis of UM Tumors

Because inactivating BAP1 mutations have emerged during the last years as prognostically relevant markers for metastatic disease,^5^ we looked for a potential correlation between histone PTMs and the BAP1 status. Of the 24 UM samples analyzed, the BAP1 status could be determined in 23 samples. Except for four tumor specimens, the BAP1 status was correctly identified using IHC. In the four discrepant cases, the result of the NGS analysis was included.

**Figure 2.**
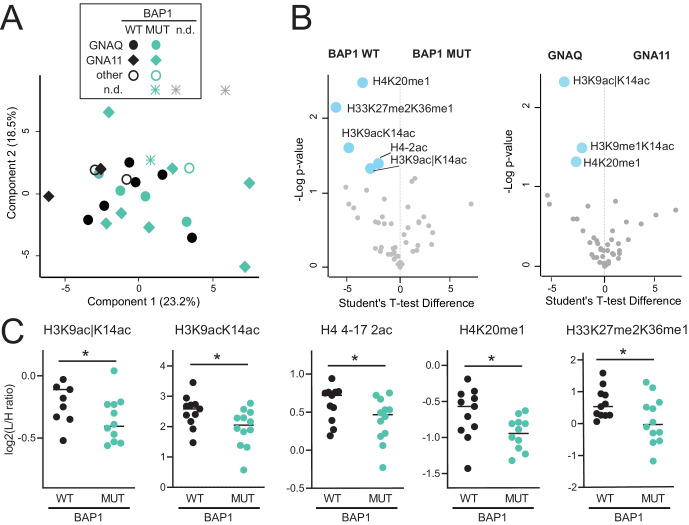
**Histone PTM profiles and mutational status in UM.** (**A**) PCA analysis based on histone PTM data obtained from the histone PTM profiling of normal choroid and UM tissues (see samples shown in Fig. 1). *Different colors* refer to BAP1 status, whereas the *symbol shapes* indicate the GNA11/QNAQ status (the *grey color* = not determined [n.d.]). (**B**) Volcano plot showing significantly up- (*red*) or down- (*blue*) regulated histone PTMs in BAP1 mutated UM (MUT) compared to BAP1 wildtype (WT) tumors (*left panel*), or GNA11 compared to GNAQ-mutated tumors (*right panel*). *P* < 0.05 by Student's *T*-test. (**C**) Display of the data for PTMs significantly changing in BAP1 mutated versus wildtype (WT) tumors. * *P* < 0.05; ** *P* < 0.01 by Student's *T*-test. The symbol “|” indicates that the modification is present on only one of the indicated residues.

Based on the hierarchical clustering, two main groups of primary UMs could be distinguished (see Fig. 1A), which, although displaying significant differences in several histone marks (see Fig. 1A, right panel), did not show a significant association with the BAP1 status. The smaller group was composed of nine tumors, six of which harbored a BAP1 mutation, whereas two tumors were BAP1 wildtype. The larger group of tumors (*n* = 15) consisted of eight BAP1-wildtype tumors and seven BAP1-mutant tumors. Although the PCA based on the histone PTM pattern also did not show a separation of samples based on the BAP1 status (Fig. 2A), five individual histone marks were associated with the BAP1 status (Figs. 2B, left panel, 2C). H3K9ac|K14ac, H3K9acK14ac, H3.3K27me2K36me1, H4 4-17 2ac, and H4K20me1 were decreased in the BAP1-mutant tumors. Of these modified peptides, H3K9ac|K14ac and H4K20me1 were also found to be decreased in GNA11-mutated tumors compared with GNAQ-mutated UM, in addition to H3K9me1K14ac (see Fig. 2B, right panel).

**Figure 3.**
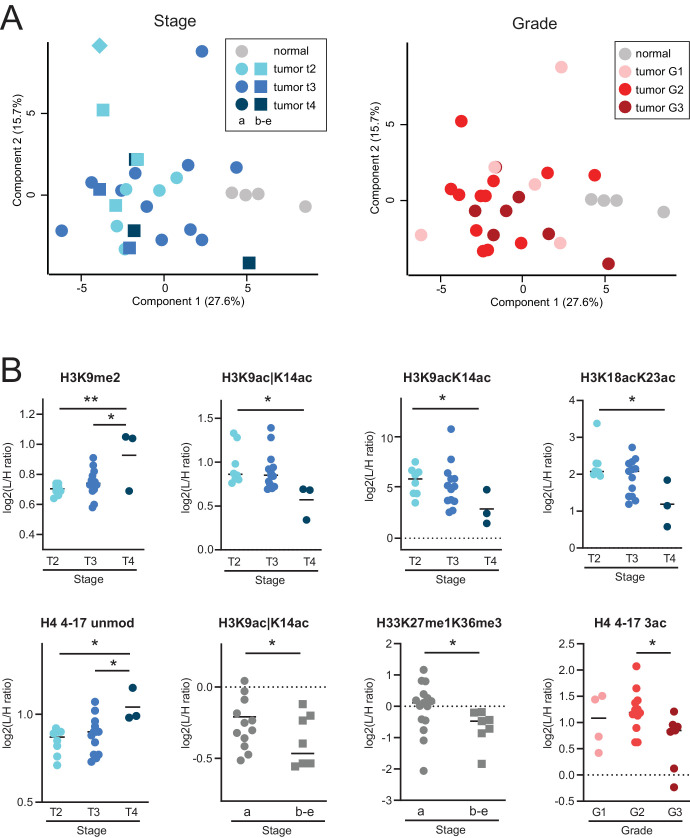
**Histone PTM profiles and staging/grading in UM.** (**A**) PCA analysis based on histone PTM data obtained from the histone PTM profiling of normal choroids and UM tissues (see samples shown in Fig. 1). *Different colors* refer to different tumor stages (*left*) or grades (*right*). (**B**) Display of the data for PTMs significantly changing in tumors with different stages and grades. * *P* < 0.05; ** *P* < 0.01 by Student's *T*-test (for the comparison of stages “**a**” versus “**b-e**”) or by 1-way ANOVA followed by a multiple comparison test (for the comparisons of tumor grades and stages 2, 3, and 4). The symbol “|” indicates that the modification is present on only one of the indicated residues.

In addition, a PCA analysis based on histone PTMs did not separate tumors according to stage or grade (Fig. 3A), although an increase in H3K9me2 and a decrease of several acetylated histone H3 peptides were associated with an increasing T stage (Fig. 3B). For example, H3K9ac│K14ac decreased with tumor stage and also in tumors with a higher risk profile (Tb-e). An increase of the unmodified form of histone H4 4-17 peptide was also observed, which indicates a parallel overall decrease of histone H4 acetylation. The tri-acetylated form of the peptide H4 4-17 was also decreased in tumors with higher grades (see Fig. 3B).

### Intratumoral Heterogeneity

**Figure 4.**
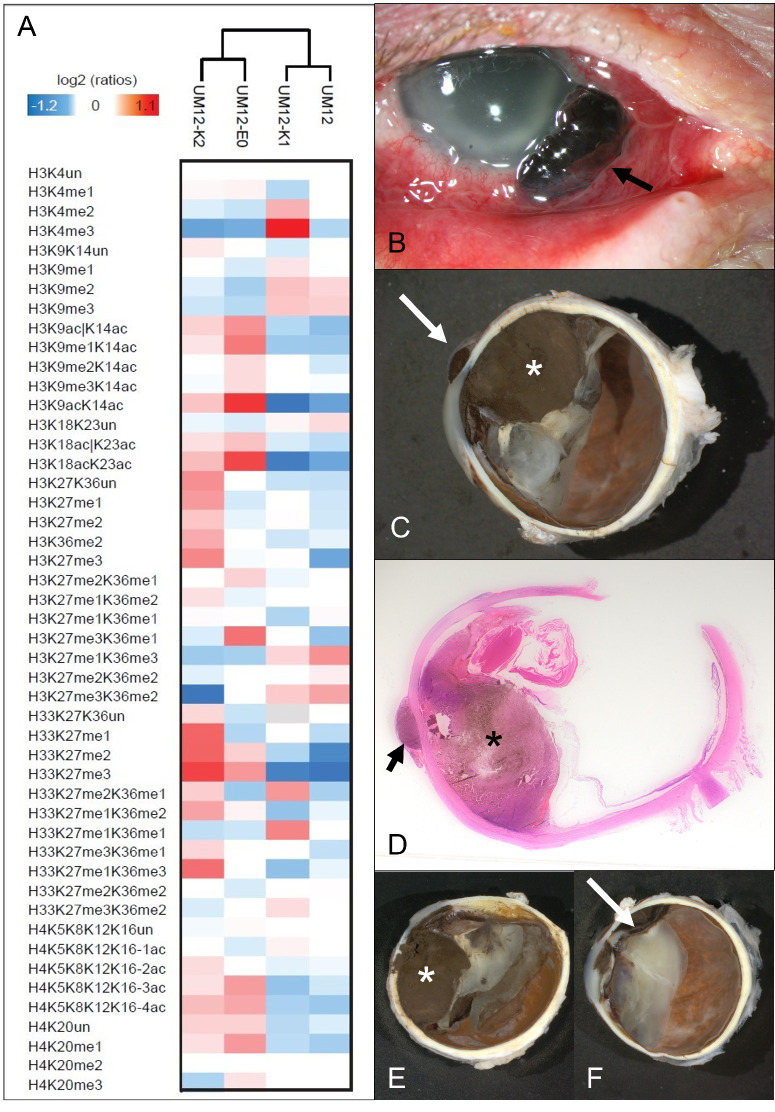
**Tumor heterogeneity.** (**A**) Heat map display of histone PTMs quantitate from different areas from UM12. Light/heavy (L/H) ratios normalized over the average ratios across samples are shown. (**B**) Clinical image of the eye with extrascleral extension (*arrow*). (**C**) Macroscopic picture of the horizontally opened globe (*asterisk* = main tumor and *arrow* = extrascleral extension). (**D**) Corresponding histopathological picture (hematoxylin-eosin stain = 4x; *asterisk* = main tumor and *arrow* = extrascleral extension). (**E**) Macroscopic picture of the inferior calotte (UM12K1) harboring a large amount of tumor (*asterisk*). (**F**) Macroscopic picture of the superior calotte (UM12K2) harboring a small amount of mainly peripheral tumor (*arrow*).

In order to assess epigenetic intratumoral heterogeneity, which may be influenced by, for example, tumor microenvironment, for one tumor sample (UM12), four different areas were analyzed (Fig. 4): the main tumor (anterior-posterior section), the inferior calotte (UM12K1, containing a large tumor part), the superior calotte (UM12K2, containing only a small peripheral area of the tumor), and the extraocular part (UM12EO, 8 × 4 mm). The four samples differed with regard to the histone PTM profile. The main tumor and the inferior calotte (UM12K1), which harbored a large amount of the main tumor, were similar for most marks, whereas the superior calotte (UM12K2) and the extraocular part of the tumor (UM12EO) differed in several epigenetic marks from the main tumor (e.g. acetylations on histone H3 K9, K14, H3K18, K23, and histone H4).

In addition, the extraocular part of the tumor showed an increase of H4K20me3, while the superior calotte showed an increase of K27 and K36 methylations on histone H3 and H3.3.

### Comparison of UM Tissues and Cell Lines

**Figure 5.**
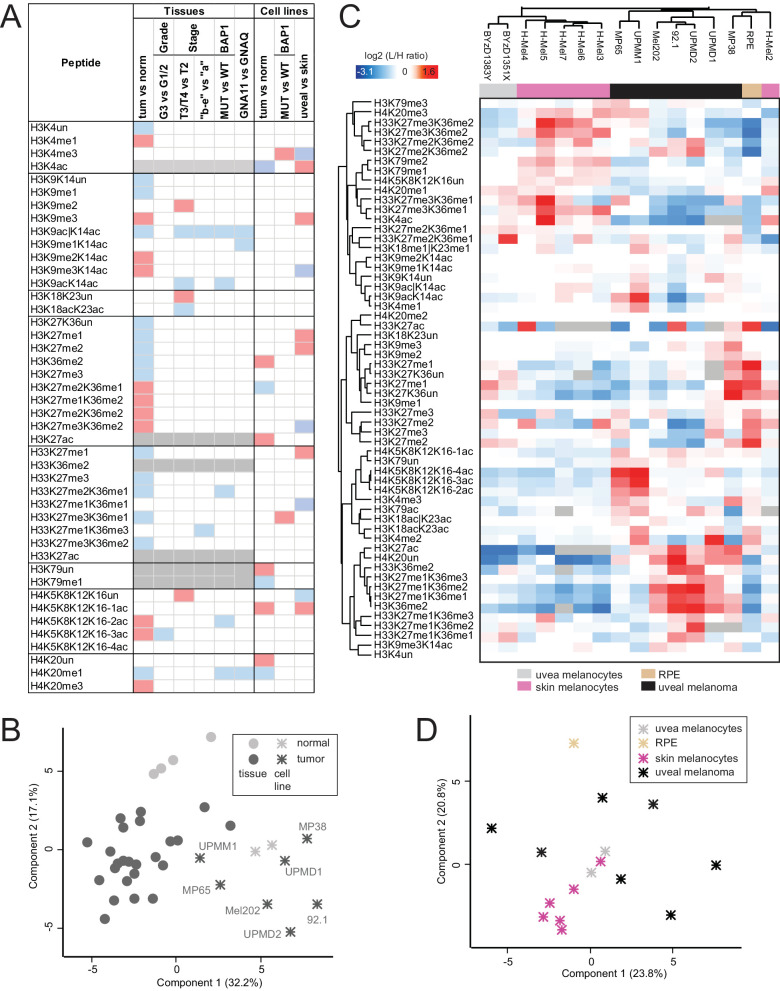
**Comparison of UM tissues and cell lines.** (**A**) Table summarizing significantly increased (*red*)/decreased (*blue*) histone modifications in UM versus normal uvea, in tumors with higher staging or grading or different mutational status. Changes in cultured skin versus uveal melanocytes are also reported. The *grey color* indicates peptides that were not analyzed in tissues. (**B**) PCA analysis based on histone PTM data obtained from the histone PTM profiling of normal choroid and UM tissues as well as uveal melanocytes and UM cell lines. (**C**) Heatmap display of histone PTM levels of healthy human uveal melanocytes and skin melanocytes as well as UM cell lines. Light/heavy (L/H) relative abundance ratios were obtained using a spike-in strategy (*light channel* = sample and *heavy channel* = spike-in standard), and were normalized over the average ratios across samples. The *grey color* indicates peptides that were not quantified. (**D**) PCA analysis based on histone PTM data obtained from the histone PTM profiling of normal uveal and skin melanocytes, and UM cell lines. RPE, retinal pigment epithelium. The symbol “|” indicates that the modification is present on only one of the indicated residues.

Because our analysis of UM tissues revealed several differences between normal/tumor tissues, among different stages and grades, and between WT and mutated BAP1 tumors (Fig. 5A), we then evaluated whether available UM cell lines could recapitulate the histone PTM patterns of UM tumors and maintain the differences observed among the different groups of tissues. Thus, we profiled seven human UM cell lines (all were of primary, non-metastatic origin) and uveal melanocytes from two donors (Table 2). In agreement with previous results,^47^ all cell lines tested clustered together and separately from the tissues (Fig. 5B), with the UPMM1 and MP65 UM cell lines clustering closer to tumor tissues (Fig. 5B). Uveal melanocytes tended to cluster together in the PCA, but the changes found in individual histone marks in UM cell lines compared with melanocytes were not always consistent with those found in tissues. However, the significant decrease in H4K20me1 in UM was confirmed in cells, and H4K20me3 showed an increasing trend, also in cell lines (*P* < 0.1; Fig. 5C).

**Table 2.** Characteristics of Analyzed Cell Lines

	Age, Y	Gender, F/M	Chromosome 3 Status	BAP1 Status	Driver Mutations
Skin melanocytes					
H-Mel 2	63	M	N/A	N/A	N/A
H-Mel 3	61	F	N/A	N/A	N/A
H-Mel 4	63	F	N/A	N/A	N/A
H-Mel 5	65	M	N/A	N/A	N/A
H-Mel 6	63	F	N/A	N/A	N/A
H-Mel 7	75	F	N/A	N/A	N/A
Uveal melanocytes					
BYzD1383Y	83	M	N/A	N/A	N/A
BYyD1351X	51	F	N/A	N/A	N/A
Uveal melanoma cell lines^41^					
92.1^35^^,^^37^^,^^38^	76	F	D3	BAP1 WT	GNAQ mut
					EIF1AX mut
UPMD1^35^^,^^36^	UKN	UKN	D3	BAP1 WT	GNA11 mut
UPMD2^35^^,^^36^	UKN	UKN	D3	BAP1 WT	GNA11 mut
					SF3B1 mut
Mel202^35^^,^^37^^,^^39^	81	F	Isodisomy 3	BAP1 WT	GNAQ mut
					SF3B1 mut
MP38^40^^,^^41^	UKN	M	Isodisomy 3	BAP1 Mut	GNAQ mut
UPMM1^35^	UKN	M	M3	BAP1 WT	GNAQ mut
MP65^40^	42	F	M3	BAP1 Mut	GNA11 mut

D3, disomy of chromosome 3; F, female; M, male; M3, monosomy of chromosome 3; Mut, mutation; N/A, not applicable; UKN, unknown; WT, wildtype.

UM cell lines with a monosomy 3 (MP65 and UPMM1) were distinguishable from cell lines with a (iso-disomy 3 [Mel202, 92.1, UPMD1, and UPMD2]). In detail, the two monosomy 3 cell lines (UPMM1 and MP65) clustered together in the PCA (Fig. 5D) which was also confirmed by hierarchical clustering (see Fig. 5C). The three disomy cell lines (UPMD1, UPMD2, and 92.1) and the isodisomy cell line Mel202 were also grouped together in the hierarchical clustering analysis. A statistically significant increase of H3K4me3 and H33K27me3K36me1 was found in UM cell lines with a BAP1 mutation. An increase of H3K27me3 was observed in the cell lines MP65 (harboring a BAP1 mutation) and – unexpectedly – UPMD1. We observed that marks for active chromatin histone modifications (H3K4ac and H3K79me1*) as well as H3K27me2K36me1* were significantly decreased in UM cell lines compared to uveal melanocytes whereas H3K36me2*, H3K27ac, and H3K79un were increased (* asterisk indicates that the identified histone modifications may be attributed to long-term culture^47^).

We also analyzed six cultured human skin melanocytes to identify differences between uveal and skin melanocytes. The analysis showed several differences between the two types of melanocytes (see Figs. 5C, 5D). Hierarchical clustering and PCA based on histone PTM patterns revealed a clear distinction between skin and uveal melanocytes (except for the skin cells named H-Mel2; see Figs. 5C, 5D). Statistically significant differences (*P* < 0.05) for histone PTM patterns between skin and uveal melanocytes were detected for 11 differentially modified peptides (see Fig. 5).

## Discussion

Studying epigenetic modifications in cancer, including UM, have gained increasing interest in recent years. An extended analysis of UM DNA methylation patterns based on the data from the “The Cancer Genome Atlas” (TCGA) project has been already performed in 2017, resulting in the identification of prognostically relevant UM subtypes.^48^ However, although histone PTMs have been extensively studied in other types of cancer, this is – to the best of our knowledge – the first systematic analysis of bulk histone PTMs in UM tissues and cell lines. The comparison of tumor tissues with control choroid revealed several altered histone PTMs, including changes in H3K9me3, H3K27, and K36 methylation, H4K20me1/me3, as well as acetylations in H3K14 and the histone H4 tails. Some of our findings are consistent with previously published data. For instance, the decrease in H3K9ac|K14ac and the increase in H3K9me3 commonly occur in cancer, whereas a decrease in H3K27me3 has been observed in triple negative breast cancer and in head and neck cancers.^49^ On the contrary, the increase observed in H4K20me3 in UM compared with normal tissues is surprising, given that the decrease of this histone mark has been reported as a general hallmark of cancer.^49^^,^^50^ However, the heat map in Figure 1 shows that the level of H4K20me3 in the tumors in one of the clusters is similar to those observed in the normal tissues and significantly lower than those of the other cluster, indicating that subgroup differences may exist. The investigation of a larger cohort of samples will help confirm whether tumor subgroups exist and whether they are characterized by specific histone PTM aberrations.

In addition, a few histone PTMs displayed changes linked to increasing tumor stage or associated with the BAP1 mutation status. Histone H3 and H4 acetylation were overall decreased in tumors with higher stages or grades, and several acetylated peptides (H3K9acK14ac, H3K9ac|K14ac, and H4 4 4-17 2ac) were decreased in BAP1-mutated tumors. In addition, we observed an association of H33K27me2K36me1 and H4K20me1 levels with a BAP1 mutation. H4K20me1, which is associated with transcriptional activation and known to be involved in DNA replication and DNA damage repair (along with H4K20me2),^51^ was reduced in UM tissue and cells, and also in BAP1-mutated tumors. H4K20me1, as well as H3K9ac|K14ac, was also decreased in GNA11-mutated compared with GNAQ-mutated tumors. Because in our cohort most of GNA11-mutated tumors also harbored a BAP1 mutation (see Table 1) it is difficult to determine whether these histone PTM changes are associated with one mutation or the other, or both. Because both BAP1^5^ and recently also GNA11^52^ have been previously reported to be associated with metastatic UM, these changes could also be linked with metastatic disease, which would be extremely interesting to investigate in a future study.

With regard to epigenetic intratumoral heterogeneity, some variation among UM samples had been previously observed for histone acetylation and DNA methylation by IHC staining in UM.^53^ Tumor heterogeneity of histone PTMs was also described in clinical cancer samples.^30^ The observed intratumoral heterogeneity may be explained by a different proportion of cells of the tumor microenvironment (mainly macrophages and T lymphocytes) in more peripheral tumor parts. Further analyses are warranted to further study histone PTM patterns in UM with spatial resolution within the tissue, in order to identify epigenetic mechanisms depending on the interaction of the tumor with the surrounding microenvironment.

The rationale underlying the use of tumor cell lines in molecular oncology is that they are a good proxy of the original tumor*,* allowing the investigation in vitro of the molecular mechanisms underpinning tumor onset, progression, and response to therapeutic agents. The epigenetic profiles of UM cell lines and melanocytes differ substantially from the tissue counterparts, which is consistent with previous reports showing that culture conditions cause multiple changes in histone PTMs.^47^ However, the UPMM1 and MP65 UM cell lines show a PTM profile closer to the primary tumor tissues, indicating that these cell lines should be preferred for in vitro experiments focused on cancer epigenetics. However, it must be noted that, in comparison to melanocyte short-term culture, the investigated tumor cell lines displayed histone mark changes that were previously associated to long-term culture,^47^ such as a decrease in H3K27me2/me3, H3K79me1/me2, and an increase in H3K36me1/me2.

There are only a few BAP1-mutated UM cell lines available and there seems to be a cell culture-associated selection for BAP1 wildtype cell lines. A BAP1 mutation has been previously associated with increased levels of H3K27me3.^11^^,^^54^^,^^55^ Although this finding is often reported, Campagne et al. did not observe a global effect of BAP1 knockout on the overall levels of H3K4me2 and H3K27me3.^56^ In our study, an increase of H3K27me3 was only observed in one of the BAP1-mutated cell lines. Thus, MP65 seems to be more representative of a BAP1-mutated tumor than MP38 (which harbors isodisomy). Because UPMD1 also showed an increased level of H3K27me3, other BAP1 wildtype cell lines should be preferred.

UM develops from uveal melanocytes, which are located in mesodermal tissues. In contrast, skin melanoma arises from epidermal melanocytes. However, both types of melanocytes originate from the same precursor cells in the neural crest.^57^^,^^58^ Whereas cutaneous and uveal melanoma differ significantly from each other with regard to the clinical behavior, the genetic profile and the response to therapy,^3^^,^^59^ molecular differences between skin and uveal melanocytes are not well characterized. The differentiation of neural crest cells into intraocular or skin melanocytes and the factors that initiate and control this process are still not fully understood.^59^ Our pilot study revealed several differences in histone marks – many of which involve methylation of H3K27 and H33K27– between uveal and skin melanocytes, consistent with the fact that UM and skin melanoma differ substantially both in their clinical behavior and in their genetic profile, prognosis, and responsiveness to various therapies. However, differences between these cell types may also be ascribed to the melanocyte environment, which is epidermal for skin melanocytes and mesenchymal (uvea containing blood vessels and other cellular components) for uveal melanocytes, as well as their neighboring cell types, for example, keratinocytes (skin melanocytes), fibroblasts, and endothelial cells (choroidal melanocytes)^60^ with which the melanocytes may interact.

In conclusion, we report in this study the first quantitative MS profiling of histone PTMs in uveal melanoma clinical samples and cell line models. Our findings indicate the presence of histone modifications with diagnostic and prognostic potential in UM, which will need to be validated in a larger cohort of patients, also in correlation to the chromosomal status of the tumors and the survival data. Further studies are also needed to characterize in depth the aspect of intratumoral epigenetic heterogeneity as well as the impact of the tumor microenvironment on histone PTMs of cancer cells. In addition, our study highlighted cell culture models that are more representative of UM and should be preferably used for the investigation of epigenetic mechanisms and epi-drug effects in vitro. Further efforts will allow to corroborate and expand this initial observation, in order to establish other in vitro models (e.g. organotypic or organoid models) that represent a better proxy for the in vivo state of UM.

## Supplementary Material

Supplement 1
